# Compliance with COVID-19 preventative health measures in the United Kingdom: a latent profile analysis

**DOI:** 10.1093/heapro/daae007

**Published:** 2024-03-02

**Authors:** Shannon Colville, Steven Lockey, Nicole Gillespie, Sarah Jane Kelly

**Affiliations:** Queensland University of Technology, School of Business, 2 George Street, Brisbane City, Queensland 4000, Australia; The University of Queensland, School of Management, Cambell Road, St Lucia, Queensland 4067, Australia; The University of Queensland, School of Management, Cambell Road, St Lucia, Queensland 4067, Australia; Queensland University of Technology, School of Business, 2 George Street, Brisbane City, Queensland 4000, Australia

**Keywords:** latent profile analysis, COVID-19 compliance, trust, vaccine uptake

## Abstract

Governments have adopted unprecedented measures to assist in slowing the spread of the COVID-19 pandemic, but compliance varies among individuals. This UK study uses latent profile analysis to identify four classes of individuals on factors believed to influence compliance. Those who sought health information from authoritative sources and actively sought information from multiple sources were most compliant. Profile differences in compliance and vaccination status were also primarily driven by trust in healthcare institutions over trust in government. These findings contribute to understanding compliance profiles and emphasise the importance of authoritative information and trust in healthcare systems.

**Contribution to Health Promotion**

- This study investigates the compliance of UK citizens with COVID-19 rules, identifying four distinct subgroups with varying levels of compliance.
- Compliance and vaccination status were primarily driven by trust in healthcare institutions over and above trust in government.
- Results highlight the importance of targeting health information and messages and show the importance of healthcare institutions in promoting pandemic compliance and shaping future health plans.

## INTRODUCTION

The coronavirus (COVID-19) pandemic was one of the most challenging public health crises of our time. While governments and public health authorities imposed strict measures to control the spread of the virus, including lockdowns, curfews, mask-wearing, social distancing regulations and mandated vaccination, community compliance was mixed (Kupferschmidt and Cohen, 2020; Martarelli and Wolff, 2020). As such, there is a pressing need to understand the complex interplay of factors that influence public compliance with government health measures to contend with COVID-19 and future health crises.

A sizable literature has emerged examining the factors influencing health compliance including vaccination status during COVID-19. This literature has identified a range of factors including perceptions and knowledge of COVID-19, political and cultural orientations, personality and sociodemographic characteristics (Blagov, 2020; lacono *et al*., 2021; Aksoy, 2022). For example, people who have more knowledge of COVID-19 and perceive it as risky are more likely to engage in compliance behaviours (Beca‐Martínez *et al.*, 2022). Individuals with collectivist values (i.e. prioritise the needs of the collective over the needs of the individual) and liberal political beliefs (characterised by values such as civic virtue, pluralism and tolerance) are more likely to engage in preventative behaviours (Bish and Michie, 2020; Wright *et al*., 2022). Regarding personality, conscientious people have been found to be more compliant than neurotic individuals (Schmeisser *et al*., 2021; Auton and Sturman, 2022). The sources individuals rely on for information can significantly influence their knowledge and attitudes towards health-related issues (Burger *et al*., 2020). For instance, Fridman *et al*. (2020) found that government information sources were the most trusted among the public, and that trust in such sources was positively associated with accurate knowledge of COVID-19 and adherence to social distancing.

Trust in institutions including government and health authorities also functions as a prerequisite to compliance behaviours (Goldstein and Wiedemann, 2020; Bicchieri *et al*., 2021). For instance, Bargain and Aminjonov (2020), Caplanova *et al*. (2021) and Shanka and Menebo (2022) found trust in public institutions positively influences compliance towards COVID-19 health measures. These studies highlight that when individuals have confidence in government institutions and health authorities, they are more likely to adhere to guidelines such as mask-wearing, social distancing and vaccination.

While these studies offer valuable insights into the factors associated with compliance and vaccination status, little is known about the interplay of these variables and how they operate together to influence compliance. This is because prior research has largely taken a linear, variable-centred approach and explored a limited range of factors in isolation, rather than considering the potentially complex relationships between them (Burger *et al*., 2020). Drawing on the discussions by Hornsey *et al*.’s (2021) of the ‘small-pockets’ problem, traditional variable-centred examinations of compliance and vaccination attitudes typically model the central tendency of a sample. Yet because a large majority of people are willing to comply with government health measures and have pro-vaccination attitudes, this approach makes it difficult to identify and understand niche groups with fringe attitudes towards compliance and vaccination.

The current study addresses these problems by adopting a person-centred approach using latent profile analysis (LPA) to identify the characteristics of subpopulations that vary in their compliance with COVID-19 health measures (Howard and Hoffman, 2017; Spurk *et al*., 2020). Our aim was to examine how a comprehensive range of factors, previously identified as influencing compliance with COVID-19 health measures, operate together in a holistic way that accounts for potential interactions between them. A strength of our person-centred LPA is its ability to handle this multidimensionality (Spurk *et al*., 2020).

Our research contributes to the literature in three ways. First, we extended previous research by exploring a comprehensive selection of variables—health information sources, knowledge and perceived risk of COVID-19, political, cultural, personality and sociodemographic factors—as profile variables. This comprehensive approach allows us to take a holistic view of the variables that have previously been recognised as influencing compliance and further explore how the various combinations of these variables affect compliance and vaccination status.

Second, we extend prior research by examining a full list of government health measures communicated by the United Kingdom (UK) Government to understand compliance behaviours in our latent profile data. Previous studies have largely focused on a small selection of government health measures, such as mask-wearing, social distancing and vaccine uptake (Chu *et al*., 2020; Kemmelmeier and Jami, 2021; Nivette *et al*., 2021). Using this large collection of compliance measures enables more nuanced inferences regarding factors that predict whether public health recommendations will be followed.

Third, drawing on institutional theory, which rests on the belief that institutions such as the government will act responsibly under normative conditions (Yuan *et al*., 2022), we examine trust in government and healthcare authorities as parallel mediators of our latent profiles’ relationships with compliance and vaccination uptake. To our knowledge, no previous studies have investigated trust in government and trust in healthcare institutions as parallel mediators between latent profiles and compliance.

### Variable selection

We chose the profiles variables, such as information source, political and cultural orientation, personality and COVID-19 risk and knowledge, based on their established significance in the literature concerning health behaviour and public health compliance, as noted in the previous section. While these factors are conceptually distinct, there are likely to be interrelations between them that are important to understand to develop a more nuanced understanding of attitudes and behaviours around COVID-19 (Gerace *et al*., 2022). LPA is an ideal method for uncovering such interrelations because it can integrate and be used to answer questions about variables that cannot easily be uncovered by traditional, regression-based analysis (Zyphur, 2009; Spurk *et al*., 2020). As an example, a recent LPA study in Singapore used a set of conceptually distinct yet interrelated set of knowledge, risk, emotional and behavioural indicators to segment and capture differences in how people respond to COVID-19 (Ong *et al*., 2023).

Trust in institutions has also been found to be an outcome of our profile variables, such as information sources (Qiao *et al*., 2020), risk (Diotaiuti *et al*., 2021), personality factors (Rammstedt *et al*., 2022), political ideology and cultural worldview (Baumgaertner *et al*., 2018; Leong *et al*., 2022) in the context of health compliance. More broadly, trust has long been recognised as having an indirect effect on health outcomes (Calnan and Rowe, 2007; Elgar, 2010), and underpinning effective health delivery (Gilson, 2003), with more recent research indicating the crucial role of trust in institutions as a mechanism for understanding how factors like health communication (Hong and Oh, 2020) and socio-psychological processes (Barattucci *et al*., 2022) impact health outcomes. For these reasons, we position trust in government and health authorities as mediators rather than profile variables.

## METHOD

### Sample

This online survey was completed by a sample of 1761 adults (18 + years) who were representative of the UK population in terms of age, sex and location. Participants were recruited by Dynata, an online panel provider, between March 17th and April 6th, 2021, when the UK was implementing government-mandated restrictions due to the COVID-19 pandemic (UK Government, 2021). Participation was voluntary and anonymous. Upon completion of the survey participants were paid $4.20. This project complied with the provisions contained in the National Statement on Ethical Conduct in Human Research (approval #2020002155). We excluded 525 respondents who failed an attention check question by responding anything other than ‘Strongly agree: to the item: “It is important that you pay attention in this study, please click Strongly agree”’. An additional 105 respondents were excluded from taking the survey implausibly quickly—less than half of the median completion time of 6 minutes. This resulted in a usable sample of 1131 respondents.

### Measures

#### Profile measures

##### Information sources.

We asked participants to rate ‘Which of the following sources do you use for information on the latest health information relating to the COVID-19 pandemic’ using a 5-point Likert scale (1 = Not at all, 5 = All the time): health experts, scientific experts and articles, government representatives, news media, social media, and people important to the respondent.

##### COVID risk and knowledge.

We measured perceived risk from COVID-19 using an adapted six-item measure from the Benthin Risk Perception Scale (Benthin *et al*., 1993). An example item was: ‘If I contract COVID-19, it will have major consequences for my health and wellbeing’ (α = 0.88). COVID-19 knowledge was measured using six items derived from the National Health Service (NHS) website (NHS, 2021). Sample items: ‘Persons with COVID-19 can infect others even when no symptoms are present’ and ‘The fatality rate is higher than the common flu’ (α = 0.81).

##### Cultural.

Six items were adopted from the Individualism-Communitarianism sub-scale of the Cultural Cognition Worldview Scale (Kahan *et al*., 2011). Three items measured individualism (sample item: ‘The government interferes far too much in our everyday lives’, α = 0.79), and three measured collectivism (sample item: ‘The government should do more to advance society’s goals, even if that means limiting the freedom and choices of individuals’, α = 0.72).

##### Political.

We adopted the following item from Piurko *et al*. (2011) to measure political orientation: ‘In politics, people sometimes talk of “left” and “right”. Where would you place yourself on a scale from 1 to 9, where 1 means extreme left and 9 means extreme right?’.

##### Personality.

Neuroticism and conscientiousness were each measured with two items from the brief Big-Five Inventory (BFI-10; Rammstedt and John, 2007). Sample items are ‘I see myself as someone who gets nervous easily’ (neuroticism) and ‘I see myself as someone who does a thorough job’ (conscientiousness). Rammstedt and John (2007) found good reliability and validity for the BFI-10 scales. We do not report internal reliability coefficients as it is not appropriate as a measure of the internal consistency of two-item measures (Eisinga *et al*., 2013). The short-form BFI-10 has been widely used, including in studies of responses to COVID-19 (Fernández *et al.*, 2020; Murphy *et al*., 2020).

#### Mediator variables

##### Trust.

We asked respondents to reflect on the COVID-19 pandemic and rate the extent to which they trust (i) the government and (ii) health authorities using a scale adapted from (Lockey *et al*., 2021). Specifically, we asked respondents to rate their trust in government and health authorities to (i) do the right thing, (ii) be transparent, (iii) operate competently, (iv) provide clear guidelines, (v) protect the health of citizens and (vi) act in the country’s best interest, on a 7-point scale (1 = Completely distrust to 7 = Completely trust). Both measures had good reliability ( = 0.97 for both).

#### Outcome variables

##### Compliance.

To measure compliance with government measures, participants rated their adherence to 14 specific behaviours, including the limit on the number of people in their homes, wearing a face mask and staying home if they are ill. These items were selected from the full list of measures provided on the UK government website at the time of data collection (see Supplementary Table S1). The items were combined into a composite scale (α = 0.87).

##### Vaccination status.

Participants were asked if they had been vaccinated, or planned to get vaccinated, against COVID-19 using four options: 0 = No, I have not been vaccinated and do not plan to be vaccinated, 1 = Unsure, I have not been vaccinated and am not sure if I will be, 2 = Yes, I plan to be vaccinated when it is my turn, 3 = Yes, I have been vaccinated against COVID-19.

#### Control variables

We included sex and education as control variables in the mediation analyses. Sex was measured as 1 = Male, 2 = Female. Age was measured as 1 = 18–25, 2 = 26–30, 3 = 31–35, 4 = 36–40, 5 = 41–45, 6 = 46–50, 7 = 51–55, 8 = 56–60, 9 = 61–65, 10 = 66–70, 11 = 71–75, 12 = 76–80, 13 = 81+. Education was measured as 1 = No qualifications, 2 = Completed secondary school to GCSE, O-Level or similar, 3 = Vocational/trade/technical qualification, 4 = Completed secondary school to A-Level or equivalent, 5 = Undergraduate degree (e.g. bachelors), 6 = Postgraduate degree (e.g. masters, PhD). We did not include these demographics as profile variables because they are likely to have a causal influence on where people seek health information, COVID-19 knowledge and risk.

## RESULTS

### Descriptive summary

Supplementary Table S1 summarises correlations, means and standard deviations of the focal variables. Noteworthy correlations include the negative relations between social media as a source of health information and both COVID-19 knowledge and vaccination status. Similarly, an individualistic worldview demonstrates negative correlations with trust in government, trust in health authorities, compliance and vaccination status, while neuroticism displays a negative correlation with vaccination status. Conversely, positive associations are evident between trust in government and both vaccination status and compliance, as well as between trust in healthcare and vaccination status and compliance.

### Profile identification and analysis

To identify latent profiles, we used Mplus 8.6 (Muthén and Muthén, 1998–2021) and included all respondents in our analyses. The rate of missing data was low, with less than 1% missing on any variable except for political ideology, where 4.4% of data were missing. To maximise generalisability (Newman, 2014) and handle the low percentage of missing data, we employed the full information maximum likelihood missing at random approach (Little and Rubin, 2019). Each analysis was run using 1000 random starting values.

Several indices can be used to help determine how many latent profiles to include in LPA, no one index should be used in isolation and the interpretability and meaningfulness of the data should be considered (Spurk *et al*., 2020). As such, we consulted the Loglikelihood statistic, the Akaike information criterion (AIC), the Bayesian information criterion (BIC), the sample-size adjusted Bayesian information criterion (SABIC), the Lo-Mendell Rubin adjusted likelihood ratio (LMRALR) statistic and entropy. Furthermore, we considered the size (%) of the smallest class and the interpretability of the solution. Smaller values for the Loglikelihood and fit statistics are indicative of good fit, and entropy values of 0.80 and above are acceptable. A significant (*p* < 0.05) LMRALR statistic indicates that the solution is a better fit to the data than a model with one classification less.

We examined the indices of a range of models; a 1-profile solution to a 7-profile solution (see Supplementary Table S2). Combining the indices and our subjective judgement of the meaningfulness and interpretability of each model, we selected the 4-profile solution as the best fit. While the 5th and 6th profiles demonstrated good entropy and marginally significant LMRALR statistics, the profile sizes were small at less than 5.5% of the sample, raising concerns about low power, lower precision relative to larger profiles and a less parsimonious model. Furthermore, new profiles generated in these solutions did not appear to be substantively different from existing profiles. Rather, they appeared to split existing profiles into extremes. Finally, examination of the elbow plot of the AIC, BIC and SABIC (see Supplementary Figure S1) indicates the last significant ‘elbow’ occurs at profile 4, providing further support for the 4-profile solution being the most optimal in this context.

### Description of profiles

**Fig. 1:**
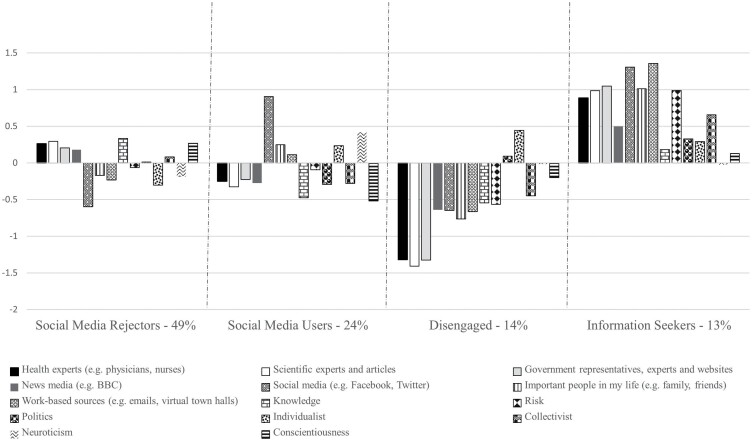
Profiles based on COVID-19 health information sources, knowledge, risk, political factors and personality.

Figure 1 shows the four profiles, including the standard deviations above and below the sample means for each of the variables to aid interpretation and allow for comparisons between profiles. The name of each profile, and the proportion of the sample it represented, is displayed under each profile. The profile names capture the variables most salient in distinguishing the profile from other profiles. We next describe each profile in turn.

Profile 1 (P1): Social media rejectors. Comprising 48.8% of the sample, P1 members have the highest vaccination rate and above-average compliance. The distinctive characteristic of this profile is its members’ reluctance to use social media for health information. They are knowledgeable about COVID-19 and tend to be slightly more collectivist and conscientious, and less neurotic than average.

Profile 2 (P2): Social media users. Comprising 23.8% of the sample, P2 members have the lowest vaccination rate and one of the lowest compliance rates. Contrary to P1, they are much more likely to get health information from social media than the sample average, which is the feature that best discriminates them from other profiles. They also have lower knowledge of COVID-19 and are more individualistic, left-wing, neurotic and less conscientious than the average.

Profile 3 (P3): Disengaged. Comprising 14% of the sample, P3 has the lowest compliance and second lowest vaccination level. This profile is characterised by disengagement and a lack of interest in seeking health information, particularly from expert sources. P3 members view COVID-19 as low-risk and have less knowledge about it. They are more individualistic and less collectivist and conscientious than the average.

Profile 4 (P4): Information seekers. Comprising 13% of the sample, P4 members have the highest compliance and one of the higher vaccination levels. This profile is distinct in seeking health information from all sources and perceiving COVID-19 as high risk. P4 is more right-leaning and collectivist than the other profiles.

Table 1 shows how each of the four profiles differs in the mean values of compliance and/or vaccination, as well as presenting the means for each of the variables used in profiling as well as the trust and control variables.

**Table 1:** Means of focal variables and demographic controls in the sample and across profiles

	Sample	Profile 1	Profile 2	Profile 3	Profile 4
	M	48.81%	23.16%	14.34%	13.52%
*Outcomes*					
Compliance	4.42	4.52	4.29	4.10	4.63
COVID-19 vaccination status	2.37	2.62	1.96	2.19	2.33
*Profile variables*					
Health information source…					
… News media	3.81	4.01	3.50	3.10	4.37
… Social media	2.20	1.42	3.39	1.36	3.91
…Government representatives, experts and websites	3.15	3.38	2.90	1.67	4.33
… Scientific experts and articles	3.23	3.57	2.86	1.61	4.37
… Work-based sources	2.27	1.99	2.41	1.46	3.94
… Important people in my life	3.10	2.91	3.37	2.25	4.21
… Health experts	3.42	3.72	3.13	1.90	4.44
Knowledge of COVID-19	6.13	6.41	5.73	5.67	6.28
Risk of COVID-19	4.73	4.65	4.61	3.96	6.08
Collectivist worldview	4.01	4.13	3.61	3.38	4.93
Individualist worldview	3.84	3.44	4.16	4.44	4.23
Political orientation	5.03	5.06	4.54	5.19	5.59
Neuroticism	3.88	3.56	4.52	3.85	3.84
Conscientiousness	5.20	5.49	4.64	4.99	5.34
*Mediators*					
Trust in government	4.37	4.46	3.82	3.88	5.49
Trust in health authorities	5.79	6.04	5.45	5.03	6.23
*Controls*					
Sex	51% female	58% male	66% female	50% female	57% female
Age	5.82	7.34	3.42	5.64	4.75
Education	4.09	4.14	4.15	3.74	4.14

As a first step to understand how profiles differ on compliance, we examine the disaggregated compliance behaviours in the whole sample and in each of the four profiles (see Supplementary Table S2). Compliance across the range of behaviours is high in our sample, ranging from avoiding touching one’s face with unwashed hands (*M* = 3.79/5) to avoiding contact with sick people (*M* = 4.75). However, there are significant differences in compliance behaviours between profiles, with small to medium effect sizes (*η*^2^ ranges from 0.02 to 0.07). P4 members are broadly the most compliant across the range of health directives, followed by P1, who were most likely to be vaccinated. In contrast, P3 members tended to be less compliant and P2 members also had low compliance and were the least likely to be vaccinated.

### Mediation analysis

Before undertaking mediation analysis, we first note that ANOVA tests indicate significant differences between profiles on trust in government (*Welch’s F* [3, 404.07] = 40.80, *p* < 0.001, *η*^*2*^ = 0.09) trust in health authorities (*Welch’s F* [3, 387.48] = 36.79, *p* < 0.001, *η*^*2*^ = 0.11), compliance (*Welch’s F* [3, 369.94] = 29.08, *p* < 0.001, *η*^*2*^ = 0.10) and vaccination status (*Welch’s F* [3, 364.71] = 45.51, *p* < 0.001, *η*^*2*^ = 0.11). Post-hoc tests indicate that P4 is more compliant than all other profiles (P2 and P3 *p*s < 0.001, P1 *p* = 0.045). P3 is less compliant than all other profiles (*p*s < 0.001), and P2 is more compliant than P3 but less so than P1 and P4 (all *p*s < 0.001). For vaccination status, P1 is more likely to be vaccinated than all other profiles (all *p*s < 0.001), P2 is less likely to be vaccinated than P1 and P4 (*p*s < 0.001) and marginally less so than P3 (*p* = 0.085). There is no difference in vaccination status between P3 and P4 (*p* = 0.50).

To investigate the differential effects of profiles on compliance and vaccination status through trust in government and trust in health authorities, we conducted a multi-categorical mediation analysis in Mplus, controlling for the influence of sex, age and education on all endogenous variables. (We also tested an alternative model where trust in government and health authorities predicted latent profiles, which in turn predicted compliance and vaccination status. The AIC and BIC values were larger for the alternative model, suggesting it was not a better fit to the data than the model we report here.) We allowed the mediators to covary with each other and also allowed the dependent variables to covary as there is a conceptual rationale to expect trust in government and trust in health authorities (Robinson *et al*., 2021), and compliance and vaccination status (Paul *et al*., 2021) to be related. As such, our hypothesised model was saturated, and no fit statistics were provided.

The use of multi-categorical independent variables requires that one group be chosen as a reference category (Hayes and Preacher, 2014). We chose P1 as the reference category to compare other profiles against because it was the largest group. Analyses were conducted using a bootstrapping method with 1000 iterations and 95% confidence intervals.

Path analysis results (see Supplementary Figure S2) indicate that P2 (government *b* =  −0.28, *SE* = 0.15, *p* = 0.027; health authorities: *b *= −0.45, *SE* = 0.10, *p* < 0.001) and P3 (government *b* = −0.46, *SE* = 16, *p* = 0.004; health authorities: *b *= −0.98, *SE* = 14, *p* < 0.001) were less likely to trust in government and health authorities than P1, whereas P4 was more likely to trust in both entities than P1 (government *b* = 1.26, *SE* = 0.15, *p* < 0.001; health authorities: *b* = 0.28, *SE* = 15, *p* = 0.004).

There were significant relationships between trust in government and compliance (*b* = 0.02, *p* = 0.017) and trust in health authorities and compliance (*b* = 0.14, *p* < 0.001). Furthermore, the relationship between trust in health authorities and vaccination status was also significant (*b* = 0.12, *p* < 0.001), but the relationship between trust in government and vaccination status was nonsignificant (*b* ≤ −0.01, *p* = 0.922).

Mediation results are displayed in Table 2, which shows the parallel indirect effects of trust in government and trust in health authorities between profile differences in compliance and vaccination status. Broadly, trust in government did not mediate relationships between profiles and either of the outcome variables. The one exception was for the path between P4 and compliance, where trust in the government fully mediated this relationship (*b* = 0.03, *p* = 0.022). This suggests that P4’s higher compliance than P1 could be explained via its greater trust in the government.

**Table 2:** Indirect effects of profiles on compliance and vaccination status via parallel mediators of trust in government and trust in health authorities

Path	*b* (95% CI)	Mediation?
*Profile 2*		
P2→Trust in government→ Compliance	−0.01 (−0.02, 0.00)	No
P2 →Trust in health authorities→ Compliance	−0.06 (−0.10, −0.04)	Yes—Partial
P2 →Trust in government → Vaccination status	0.00 (−0.01, 0.01)	No
P2 →Trust in health authorities → Vaccination status	−0.06 (−0.10, −0.04)	Yes—Partial
*Profile 3*		
P3 →Trust in government→ Compliance	−0.01 (−0.02, 0.00)	No
P3 →Trust in health authorities→ Compliance	−0.13 (−0.20, −0.08)	Yes—Partial
P3 →Trust in government → Vaccination status	0.00 (−0.01, 0.01)	No
P3 →Trust in health authorities → Vaccination status	−0.12 (−0.19, −0.07)	Yes—Full
*Profile 4*		
P4 →Trust in government→ Compliance	0.03 (0.01, 0.07)	Yes—Full
P4 →Trust in health authorities→ Compliance	0.04 (0.01, 0.07)	Yes—Full
P4 →Trust in government → Vaccination status	−0.00 (−0.04, 0.03)	No
P4 →Trust in health authorities → Vaccination status	0.04 (0.01, 0.07)	Yes—Full

*Note.* P = Profile (e.g. P2 = Profile 2). Unstandardised coefficients are displayed. All profiles are compared against Profile 1.

In contrast to trust in government, trust in health authorities played a consistent mediating role across all profiles. Trust in health authorities partially mediated the relationships between P2 and compliance (*b* = −0.06, *p* = 0.018) and P2 and vaccination status (*b* = −0.06, *p* = 0.003). These results indicate that P2’s lower compliance and likelihood to trend towards vaccine hesitancy, compared to P1, can be partially attributed to its lower trust in health authorities. Trust in health authorities also partially mediated the relationship between P3 and compliance (*b* = −0.13, *p* = 0.006), and fully mediated the relationship between P3 and vaccination status (*b* = −0.12, *p* ≤ 0.001). Finally, trust in health authorities fully mediated the relationships between P4 and compliance (*b* = 0.04, *p* = 0.006) and P4 and vaccination status (*b* = 0.04, *p* = 0.015). These results indicate that P4’s comparatively higher compliance and vaccination status than P1’s occur via higher levels of trust in health authorities.

## DISCUSSION

The goal of this study was to examine how subgroups within the UK population differ in their compliance with COVID-19 health measures based on an integrative selection of characteristics including health information sources, knowledge and perceived risk of COVID-19, political, personality and sociodemographic factors. While we found that the sample had high compliance with the government’s health measures, four profiles of individuals with differential levels of compliance and vaccination status emerged. The largest profile had the highest vaccine uptake and second-highest level of compliance and was characterised by a reluctance to seek health information from social media. The least compliant profile was characterised by a disengagement from seeking COVID-19-related health advice from all sources, low levels of COVID-19 knowledge and perceived risk and had a more individualistic orientation. Furthermore, the mediation analysis demonstrated that trust in healthcare institutions played a pivotal role in explaining the differences in compliance and vaccine status among these groups.

Our results show the importance of health promotion messages for informing and promoting health measures during the COVID-19 pandemic. Profiles of individuals who sought information from authoritative sources or actively sought information from all sources demonstrated greater knowledge about COVID-19, its associated risks and had higher compliance and vaccination rates compared to those who primarily relied on social media for health information or were not engaged in seeking health information from any source. These findings support the emerging research on the linkages between information sources and knowledge (Burger *et al*., 2020; Pavelea *et al*., 2021).

Findings also complement research on the role of personality traits and cultural orientation (Biddlestone *et al*., 2020; Kemmelmeier and Jami, 2021). In line with previous research, we find that profiles in which people are more likely to adhere to government health measures and be vaccinated are more collectivist than individualist. Findings also indicate that profiles in which people are more conscientious than neurotic are more likely to comply and be vaccinated. This is consistent with previous studies and supports the idea that people with a conscientious personality are more likely to follow rules, regulations and instructions (Blagov, 2020; Han, 2021; Lu *et al*., 2021).

Results related to political orientation were broadly contrary to previous work, contradicting the trend of regression-based findings that conservatives tend to be slightly more vaccine-hesitant than liberals (e.g. Baumgaertner *et al*., 2018). The most left-wing profile in our analysis was the least likely to be vaccinated and was less compliant with government directives than the most right-wing profile. The left-wing profile’s lower compliance could be attributed to the incumbent right-wing government in the UK. Furthermore, previous work has indicated that people with strong liberal tendencies may be vaccine-hesitant (Hornsey *et al*., 2021). While we did not find a strong left-wing, overtly vaccine-skeptical profile like Hornsey and colleagues, our results do not support previous findings that conservatives are more vaccine-skeptical.

The results of our study also indicate that trust in public health institutions plays a greater role in promoting compliance behaviours than trust in government. Our findings reveal the important role trust in health authorities plays in mediating the relationship between different profiles and compliance behaviours. While trust in the government had little impact on compliance, trust in health authorities was a crucial factor in promoting compliance and willingness to be vaccinated. For example, P4 had greater compliance and vaccination rates due to their high trust in health authorities, while P3’s lower vaccination willingness is linked to lower trust compared to P1. Trust in health authorities partially mediated all other relationships between profiles and outcomes. Trust in government only mediated one relationship: that of P4 and compliance. This may not be surprising given the UK government at the time of data collection was right-wing, and P4 was the most right-leaning.

### Practical implications

Our findings build upon existing research relating to the COVID-19 pandemic and compliance by bridging the gap between social and health sciences (Dror *et al*., 2020; Jiang and Dodoo, 2021; Pereira and Stornelli, 2022). The granular segmentation we propose based on our findings has several implications for health promotion and communication. First, our findings suggest that health promotional communications need to target population segments. These segments are contingent largely upon the source of health information and the propensity to trust the information. Second, our research highlights the need for early and targeted educational campaigns delivered through trusted mediums, and that the degree of trust will vary across the population so that a ‘one-size-fits-all’ message will not be as effective as a more customised message directed towards the segments identified by our research. Our robust profiling of relevant segments will assist efficiency, reach and effectiveness of health promotional campaigns and, specifically, the deployment of targeted and timely educational campaigns directed towards the vaccine-hesitant vulnerable segments, whose personal profile reflects a propensity to succumb to misinformation or low knowledge of the pandemic (Jiang and Dodoo, 2021; Pereira and Stornelli, 2022). As the pandemic has revealed inconsistent compliance with vaccines and other health protectionist mandates, governments must develop effective promotions designed to resonate with specific segments most at risk. Third, our research provides empirical data to support the efficient identification and targeting of sub-groups of the population who may be most at risk and helps to guide relevant customised communications to different segments.

### Limitations and future directions

A limitation of the study is that our data are cross-sectional. While we believe there is a conceptual rationale to expect profiles to influence trust in institutions rather than trust in institutions influencing profiles, and this directionality is a better fit to our data than the alternative, the causality of the proposed relationships between profiles, trust in institutions and compliance outcomes cannot be assumed. Future studies could employ longitudinal research designs to test this proposition and explore dynamic shifts in compliance and the stability of the latent profiles. Additionally, the data from this study were collected in one country, and caution should be exercised in generalising the findings to other countries. Future studies should determine if different profiles emerge across countries, cultures and crises. A final limitation is our use of short, two-item measures of conscientiousness and neuroticism due to concerns about time constraints. While the short-form BFI-10 has been validated as an acceptable alternative to the 44-item BFI, it displays less validity and reliability compared to the full-scale version (Rammstedt and John, 2007).

## CONCLUSION

The study examined compliance with government health measures to combat COVID-19 in the UK amongst different groups of people. The results emphasise the important role that healthcare authorities play in promoting behaviour change through evidence-based communication strategies. To be effective, these strategies should be tailored to the specific characteristics of the different groups and delivered through appropriate information channels to reach diverse groups including those who are resistant to expert and traditional media sources.

## Supplementary Material

daae007_suppl_Supplementary_Tables_S1-S2_Figures_S1-S2
