# Supplementary material for: Compliance with COVID-19 preventative health measures in the United Kingdom: a latent profile analysis
Source: Health Promot Int. 2024 Mar 2;39(2):daae007. doi: 10.1093/heapro/daae007 (PMC10908350; doi:10.1093/heapro/daae007)

**Supplementary Tables and Figures**

Table S1. Means, standard deviations and correlations between focal variables.

| Variable | M | SD | 1 | 2 | 3 | 4 | 5 | 6 | 7 | 8 | 9 | 10 | 11 | 12 | 13 | 14 | 15 | 16 | 17 |
| --- | --- | --- | --- | --- | --- | --- | --- | --- | --- | --- | --- | --- | --- | --- | --- | --- | --- | --- | --- |
| 1. Compliance | 4.42 | 0.55 | -- |  |  |  |  |  |  |  |  |  |  |  |  |  |  |  |  |
| 2. Vaccination status | 2.37 | 0.81 | .23 | -- |  |  |  |  |  |  |  |  |  |  |  |  |  |  |  |
| 3. News media | 3.81 | 1.12 | .23 | .22 | -- |  |  |  |  |  |  |  |  |  |  |  |  |  |  |
| 4. Social media | 2.23 | 1.28 | -.02 | -.30 | .03 | -- |  |  |  |  |  |  |  |  |  |  |  |  |  |
| 5. Government representatives | 3.14 | 1.13 | .27 | .10 | .26 | .20 | -- |  |  |  |  |  |  |  |  |  |  |  |  |
| 6. Scientific experts | 3.27 | 1.17 | .24 | .15 | .25 | .10 | .57 | -- |  |  |  |  |  |  |  |  |  |  |  |
| 7. Work-based sources | 2.28 | 1.23 | .07 | -.10 | .10 | .41 | .34 | .29 | -- |  |  |  |  |  |  |  |  |  |  |
| 8. Important people in my life | 3.10 | 1.11 | .08 | -.08 | .13 | .40 | .26 | .21 | .37 | -- |  |  |  |  |  |  |  |  |  |
| 9. Health experts | 3.42 | 1.15 | .27 | .17 | .22 | .11 | .42 | .60 | .30 | .35 | -- |  |  |  |  |  |  |  |  |
| 10. COVID-19 knowledge | 6.13 | 0.84 | .45 | .34 | .35 | -.12 | .23 | .26 | -.03 | .02 | .24 | -- |  |  |  |  |  |  |  |
| 11. COVID-19 risk | 4.73 | 1.36 | .35 | .09 | .15 | .20 | .23 | .23 | .26 | .26 | .32 | .19 | -- |  |  |  |  |  |  |
| 12. Individualistic | 3.84 | 1.35 | -.24 | -.23 | -.19 | .17 | -.14 | -.10 | .06 | .06 | -.10 | -.28 | -.01 | -- |  |  |  |  |  |
| 13. Collectivistic | 4.01 | 1.41 | .20 | .16 | .16 | .08 | .26 | .16 | .20 | .12 | .23 | .20 | .24 | -.19 | -- |  |  |  |  |
| 14. Political orientation | 5.03 | 1.68 | -.02 | .07 | .05 | -.05 | .10 | .00 | .08 | .04 | .04 | -.04 | .08 | .04 | .16 | -- |  |  |  |
| 15. Conscientiousness | 5.20 | 1.19 | .22 | .14 | .10 | -.14 | .12 | .15 | -.02 | -.04 | .12 | .24 | .04 | -.08 | .04 | .08 | -- |  |  |
| 16. Neuroticism | 3.87 | 1.55 | -.05 | -.17 | -.05 | .16 | -.03 | -.06 | .07 | .10 | -.06 | -.07 | .09 | .06 | -.02 | -.16 | -.27 | -- |  |
| 17. Trust in government | 4.37 | 1.72 | .24 | .18 | .22 | .02 | .33 | .13 | .16 | .18 | .23 | .17 | .28 | -.20 | .32 | .48 | .08 | -.15 | -- |
| 18. Trust in health | 5.79 | 1.22 | .39 | .28 | .31 | -.03 | .23 | .26 | .06 | .11 | .37 | .49 | .24 | -.26 | .25 | .03 | .19 | -.10 | .39 |

*Note*. *ns* range from 1081-1131. Variables 3 to 9 are sources from which people get their COVID-19 health information. Correlations equal to or greater than .06, .08, and .10 are significant at .05, .01, and .001 respectively.

**Table S2. Classification indices for a range of profile models (from 1 to 7 classes)**

| Model | Log Likelihood | AIC | BIC | SABIC | Entropy* | Smallest Class % | LMRALR** *p* |
| --- | --- | --- | --- | --- | --- | --- | --- |
| 1 | -25741 | -- | -- | -- | -- | -- | -- |
| 2 | -25066 | 50218 | 50434 | 50297 | 0.74 | 36.7 | 0.009 |
| 3 | -24645 | 49407 | 49698 | 49514 | 0.85 | 14.14 | <.001 |
| 4 | -24415 | 48977 | 49344 | 49112 | 0.85 | 13.35 | <.001 |
| 5 | -24279 | 48734 | 49177 | 48897 | 0.82 | 5.48 | 0.092 |
| 6 | -24145 | 48496 | 49015 | 48687 | 0.86 | 5.22 | 0.073 |
| 7 | -24045 | 48327 | 48921 | 48546 | 0.81 | 5.31 | 0.261 |

**Note.** AIC=Akaike Information Criteria; BIC=Bayesian Information Criteria; SABIC=Sample Size Adjusted Akaike Bayesian Information Criteria (generally lower numbers indicate stronger evidence for the classification), LMRALR = Lo-Mendell Rubin Adjusted Likelihood Ratio.

*Higher entropy is considered to reflect a more accurate representation of the existence of a particular classification solution; as entropy approaches 1, this indicates a better quality of classification (the classification is considered less accurate when the number falls lower than 0.80).

**Where the *p*-value is < .05 on any given classification profile, it is considered evidence that this profile fits the data better than a solution with one less profile.

**Figure S1: The elbow plot of the information criteria for the latent profile analysis**


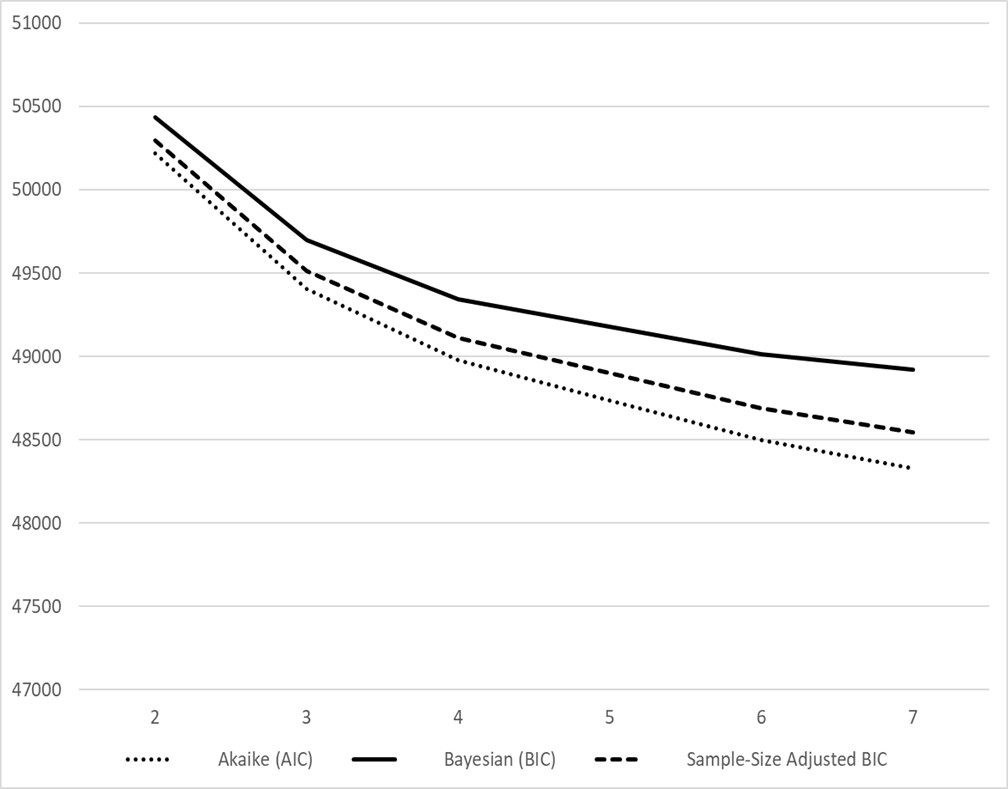


**Figure S2: Path Model**


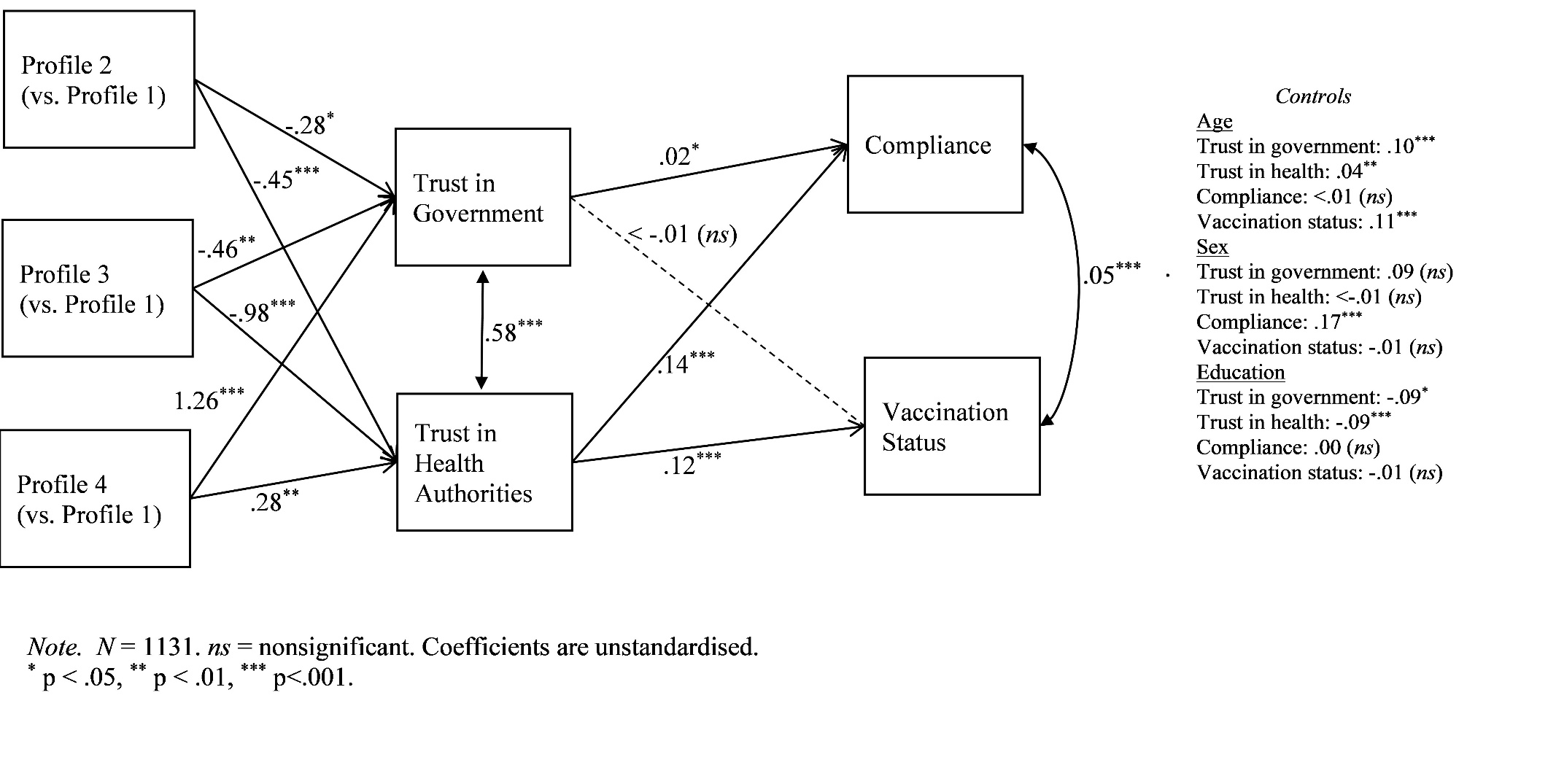

Supplement: daae007_suppl_Supplementary_Tables_S1-S2_Figures_S1-S2 [file daae007_suppl_supplementary_tables_s1-s2_figures_s1-s2.docx]
